# Evaluation of health recommender systems: a scoping review protocol

**DOI:** 10.1136/bmjopen-2023-083359

**Published:** 2024-10-07

**Authors:** Ananya Ananthakrishnan, Madison Milne-Ives, Rohit Shankar, Edward Meinert

**Affiliations:** 1 Translational and Clinical Research Institute, Newcastle University, Newcastle upon Tyne, UK; 2 Centre for Health Technology, School of Nursing and Midwifery, University of Plymouth, Plymouth, Devon, UK; 3 Plymouth University Peninsula Medical School, Truro, UK; 4 CIDER, Cornwall Partnership NHS Foundation Trust, Truro, UK; 5 Department of Primary Care and Public Health, School of Public Health, Imperial College London, London, UK; 6 Faculty of Life Sciences and Medicine, King’s College London, London, UK

**Keywords:** Artificial intelligence, eHealth, Health informatics, Decision making, Self-management, Review

## Abstract

**Abstract:**

**Background:**

People increasingly rely on online health information for their health-related decision-making. Given the overwhelming amount of information available, the risk of misinformation is high. Health recommender systems, which recommend personalised health-related information or interventions using intelligent algorithms, have the potential to address this issue. Many such systems have been developed and evaluated individually, but there is a need to synthesise the evaluation findings to identify gaps and ensure that future recommender systems are designed to have a positive impact on health or target behaviours.

**Objective:**

The purpose of this review is to provide an overview of the state of the literature evaluating health recommender systems and highlight lessons learnt, methodological considerations and gaps in current research.

**Methods and analysis:**

The review will follow the Preferred Reporting Items for Systematic Reviews and Meta-Analyses extension for Scoping Reviews and the Population, Concept, and Context frameworks. Five databases (PubMED, ACM Digital Library Full-Text Collection, IEEE Xplore, Web of Science and ScienceDirect) will be searched for studies published in English that evaluate at least one health recommender system using search terms following the themes reflecting digital health, recommendation systems and evaluations of efficacy and impact. After using EndNote 21 for initial screening, two independent reviewers will screen the titles, abstracts and full texts of the references, and then extract data from included studies related to the recommender system characteristics, evaluation design and evaluation findings into a predetermined form. A descriptive analysis will be conducted to provide an overview of the literature; key themes and gaps in the literature will be discussed.

**Ethics and dissemination:**

Ethical approval is not required as data will be obtained from already published sources. Findings from this study will be disseminated via publication in a peer-reviewed journal.

**STRENGTHS AND LIMITATIONS OF THIS STUDY**

- This review will include the search of five databases that comprehensively cover the health and technology fields to reduce the risk of excluding relevant studies.
- This study will be rigorous and strictly adhere to the Preferred Reporting Items for Systematic Reviews and Meta-Analyses extension for Scoping Reviews framework.
- The review will not search for grey literature to keep the scope manageable and ensure the quality of studies explored, but this may lead to potentially relevant research being overlooked.
- A large amount of heterogeneity in recommender types and outcome measures is expected, which might make comparison across studies more difficult.

## Introduction

 The internet is increasingly being used to seek out health information and has gone a long way in making such knowledge more accessible to the public. For example, Google reported receiving over 1 billion health-related questions per day in 2019 with 7% of all daily searches being health-related.^1^ Studies indicate that this readily available online information may have a substantial impact on people’s health-related decision-making and is capable of alleviating their health-related anxiety.^2 3^ A barrier to these potential benefits, however, is that the vast quantity of available information might lead to information overload.^4^ The nature of the information found also has the potential to cause considerable anxiety.^5^ Additionally, online media is often unregulated, leading to a risk of misinformation and potential harm.^3^ These issues underscore a clear need for effective systems that can sort out the information and offer trustworthy recommendations of information or actions based on reliable sources and a strong body of evidence.

Health recommender systems (HRSs), which use intelligent algorithms to recommend personalised information, resources or interventions relevant to the user’s specific health needs,^6^ are now available to address this need.^7^,^14^ In recent years, there has been an increase in the use, development and evaluation of HRSs. Several studies, using different methodologies, have evaluated HRSs and found mixed results. Some studies showed improved outcomes and increased user engagement,^8^,^10^ while others showed no difference in outcomes compared with waitlist controls.^11^ The quality of the studies evaluating the recommender systems has also been mixed. Some studies have been randomised controlled trials,^11 12 15^ some were single-session studies that asked users to rate recommendations after one use^9 16^ and others did not involve the users directly, instead opting to evaluate their systems using real-world datasets.^13 14^ Given the substantial potential impact of HRS information on people’s health choices, knowledge of efficacy and positive impact related to the HRSs is essential, as inaccurate or ineffective systems may be more detrimental than beneficial.

Previous reviews have synthesised the technical aspects of HRSs, focusing on the specific algorithms used or discussing the design of these systems.^17 18^ Others have provided a broad overview of the state of the literature and highlighted common methodological limitations (such as small sample size) in evaluations of HRSs.^19 20^ The gap in the existing reviews is an examination of the evaluations of existing health recommender systems and their findings in terms of outcome improvement, accuracy of recommendations and user engagement. A search on PROSPERO using the keywords (“recommendation systems” OR recommender OR personalis* OR personaliz*) AND (health OR well-being OR wellbeing) AND (digital OR ai OR “artificial intelligence”) AND (effectiveness OR efficacy OR improve*) found several planned reviews exploring user engagement or effectiveness of HRSs. These reviews have a narrow focus on specific health behaviours, health conditions, patient groups or a combination of these; none provide an overview of the state of the field of HRSs.

Given the amount and variety of the evaluations of existing HRSs, a systematic scoping review is needed to summarise the available evidence; to identify key themes, strengths and limitations of the existing research; and to examine any factors that might influence their impact (eg, type of algorithm used, clinical area, type of recommendation provided).^21^ Such an overview will help inform the design and evaluations of any such systems in the future.

This scoping review seeks to answer the research question: What is the evidence derived from evaluations of existing digital health recommender systems? To answer this research question, there will be three key objectives: (1) to synthesise the findings from evaluations of existing HRSs in terms of outcome improvement, accuracy of recommendations and user engagement; (2) to explore the factors influencing the impact of HRSs and (3) to evaluate the strengths and limitations of the existing literature on this topic.

## Methods and analysis

### Overview

The Preferred Reporting Items for Systematic Reviews and Meta-Analyses extension for Scoping Reviews (PRISMA-ScR; see online supplemental appendix A for the PRISMA-ScR checklist)^22^ and Population, Concept, and Context (PCC; see table 1)^23^ frameworks were used to structure this review and develop the search strategy.

**Table 1:** Population, Concept, and Context framework

Population	Patients (all ages) with different physical or mental health conditions.
Concept	Evaluations of digital recommender systems that aim to improve the health/well-being of participants.
Context	Any context.

### Search strategy

The search will be conducted in the following five databases: PubMED, ACM Digital Library Full-Text Collection, IEEE Xplore, Web of Science and ScienceDirect. These databases were chosen because they were commonly used in previous reviews of HRSs and comprehensively cover the health and digital technology fields. Relevant keywords and MeSH terms were identified in a preliminary literature search and examination of previous reviews conducted on HRSs and expanded to develop the search strategy. The terms were grouped into four themes to develop a string structure as follows: health (MeSH OR Keywords) AND recommendation system (Keywords) AND digital (MeSH OR Keywords) AND effectiveness (Keywords) (table 2; see online supplemental appendix B for sample search strings). In addition, the bibliographies of relevant reviews will be manually screened to identify any additional papers.

**Table 2:** Search string

Category	MeSH	Keywords (in title or abstract)
Health	“Health” OR “Delivery of Health Care”	health OR patient* OR wellbeing OR well-being OR healthcare
Recommendation system	No relevant MeSH terms identified	“recommendation system” OR “recommendation systems” OR “recommender system” OR “recommender systems” OR recommender OR “recommendation service” OR personaliz* OR personalis*
Digital	“Algorithms” OR “Telemedicine” OR “Mobile Applications”	Digital OR algorithm OR “artificial intelligence” OR “machine learning” OR ai OR online OR web-based OR internet-based OR app OR apps OR mhealth OR “mobile application*” OR “smartphone application*” OR “iphone application*”
Effectiveness	No relevant MeSH terms identified	Effective OR effectiveness OR improve* OR evaluat* OR efficacy OR accuracy OR accurate

* Refers to the symbol commonly used by databases to indicate truncation or ‘wildcard’ searching, which has been used to indicate a search of all variants of the mentioned terms.

### Inclusion criteria

This review will include studies that evaluate at least one HRS. The HRSs evaluated must apply personalisation techniques (ie, the recommendations are based on user information and are personalised to meet each specific user’s health needs) and use digital technology to generate and deliver recommendations. Studies with patients of any age (children or adults) will be eligible for inclusion. All types of study designs will be included as long as they are evaluations of one or more HRSs. In addition, studies will not be restricted based on the type of recommendation provided or type of health condition of the patients to ensure that all eligible studies are captured.

### Exclusion criteria

Studies that do not evaluate one or more health recommender systems (eg, protocols, reviews, meta-analyses, descriptions of design or development) will be excluded from the review. Studies of HRSs where the recommendation(s) were provided manually and not through an algorithm (eg, HRSs that involve clinician input during use) will also be excluded. Studies with clinicians as the target user (eg, an HRS that helps doctors personalise interventions) will be excluded. Finally, studies that are not published in English and those for which the full-text cannot be accessed will also be excluded.

### Article screening and selection

The references will be exported and stored in the citation management software EndNote 21, which will be used to identify and remove duplicates. The screening will then be done in three phases. Keywords based on the inclusion and exclusion criteria will be entered into EndNote 21’s search function over multiple passes to automatically exclude ineligible studies such as reviews and protocols. The titles and abstracts and then the full texts of the remaining papers will then be screened by two independent reviewers. The reasons for exclusion will be recorded at the full-text screening stage. Any difference of opinion at either stage of screening will be discussed until consensus; if an agreement cannot be reached, a third reviewer will be consulted. To ensure transparency and replicability, the details of the screening will be recorded in a PRISMA flow diagram, and the EndNote 21 searches will be recorded separately in an Appendix.

### Data extraction

The two reviewers will read the full texts of all the included papers and extract predetermined data (table 3). As with the screening, any disagreements will be discussed and resolved by consulting a third reviewer if necessary.

**Table 3:** Data to be extracted

Article information	Year of publication Country of study Study design Sample size Sample demographics (age, gender, target population) Study duration
Intervention	Name of recommender system Health condition(s)/behaviour(s) targeted Type of recommendations provided (information, action, other resources)
Evaluation	Evaluation methodology Outcomes measured Main findings (ie, accuracy, improved health outcomes, user engagement, drop-out rates) Participant satisfaction/perspectives (if reported) Strengths of study Limitations/weaknesses of study

### Data analysis and synthesis

A variety of HRS types, evaluation designs and outcome measures are expected, so a descriptive analysis will be used to provide a summary of the research being conducted, including the outcomes evaluated, evidence of impact, the strengths and limitations of the studies and any gaps for future research to focus on. Specific analyses related to the target health conditions/behaviours will depend on the data found in the review. For example, sub-analyses will compare the data collected across categories such as the type of HRS or the type of health condition targeted. If applicable, thematic synthesis will be conducted to generate insights from qualitative data about participant experiences with the HRS. Factors influencing the impact of HRSs will also be explored and synthesised.

### Planned timeline of the review

The literature search began soon after the submission of this protocol in December 2023 and the review is complete as of July 2024.

### Ethics and dissemination

This study will not involve human participation, and all the data for the review will be obtained from publicly available sources. As such, no ethical approval is required for this study. Dissemination of the scoping review will include publication in a peer-reviewed journal and may also include presentation of the results at relevant conferences.

## supplementary material

10.1136/bmjopen-2023-083359 online supplemental file 1

10.1136/bmjopen-2023-083359 online supplemental file 2
