# Supplementary material for: Evaluation of health recommender systems: a scoping review protocol
Source: BMJ Open. 2024 Oct 7;14(10):e083359. doi: 10.1136/bmjopen-2023-083359 (PMC11459321; doi:10.1136/bmjopen-2023-083359)
Supplement: online supplemental file 2 [file bmjopen-14-10-s002.pdf]

Appendix B: Sample Search Strings

Database	Search String	Results
PubMed	(((health[Title/Abstract] OR patient*[Title/Abstract] OR wellbeing[Title/Abstract] OR well-being[Title/Abstract] OR healthcare[Title/Abstract] OR "Health"[Mesh] OR "Delivery of Health Care"[Mesh]) AND ("recommendation system"[Title/Abstract] OR "recommendation systems"[Title/Abstract] OR "recommender system"[Title/Abstract] OR "recommender systems"[Title/Abstract] OR recommender[Title/Abstract] OR "recommendation service"[Title/Abstract] OR personaliz*[Title/Abstract] OR personalis*[Title/Abstract])) AND (Digital[Title/Abstract] OR algorithm[Title/Abstract] OR "artificial intelligence"[Title/Abstract] OR "machine learning"[Title/Abstract] OR ai[Title/Abstract] OR online[Title/Abstract] OR web-based[Title/Abstract] OR internet-based[Title/Abstract] OR app[Title/Abstract] OR apps[Title/Abstract] OR mhealth[Title/Abstract] OR "mobile application*"[Title/Abstract] OR "smartphone application*"[Title/Abstract] OR "iphone application*"[Title/Abstract] OR "Algorithms"[Mesh] OR "Telemedicine"[Mesh] OR "Mobile Applications"[Mesh])) AND (Effective[Title/Abstract] OR effectiveness[Title/Abstract] OR improve*[Title/Abstract] OR evaluat*[Title/Abstract] OR efficacy[Title/Abstract] OR accuracy[Title/Abstract] OR accurate[Title/Abstract])	8,545[a]
ACM Digital Library Full-text Collection[b]	[[Title: health] OR [Title: patient*] OR [Title: wellbeing] OR [Title: "well-being"] OR [Title: healthcare] OR [Title: "delivery of health care"] OR [Abstract: health] OR [Abstract: patient*] OR [Abstract: wellbeing] OR [Abstract: "well-being"] OR [Abstract: healthcare] OR [Abstract: "delivery of health care"]] AND [[Title: "recommendation system"] OR [Title: "recommendation systems"] OR [Title: "recommender system"] OR [Title: "recommender systems"] OR [Title: recommender] OR [Title: "recommendation service"] OR [Title: personaliz*] OR [Title: personalis*] OR [Abstract: "recommendation system"] OR [Abstract: "recommendation systems"] OR [Abstract: "recommender system"] OR [Abstract: "recommender systems"] OR [Abstract: recommender] OR [Abstract: "recommendation service"] OR [Abstract: personaliz*] OR [Abstract: personalis*]] AND [[Title: digital] OR [Title: algorithm] OR [Title: algorithms] OR [Title: "artificial intelligence"] OR [Title:	3,341[a]

	"machine learning"] OR [Title: ai] OR [Title: online] OR [Title: "web-based"] OR [Title: "internet-based"] OR [Title: app] OR [Title: apps] OR [Title: mhealth] OR [Title: "mobile application*"] OR [Title: "smartphone application*"] OR [Title: "iphone application*"] OR [Title: telemedicine] OR [Abstract: digital] OR [Abstract: algorithm] OR [Abstract: algorithms] OR [Abstract: "artificial intelligence"] OR [Abstract: "machine learning"] OR [Abstract: ai] OR [Abstract: online] OR [Abstract: web-based] OR [Abstract: internet-based] OR [Abstract: app] OR [Abstract: apps] OR [Abstract: mhealth] OR [Abstract: "mobile application*"] OR [Abstract: "smartphone application*"] OR [Abstract: "iphone application*"] OR [Abstract: telemedicine]] AND [[Title: effective] OR [Title: effectiveness] OR [Title: improve*] OR [Title: evaluat*] OR [Title: efficacy] OR [Title: accuracy] OR [Title: accurate] OR [Abstract: effective] OR [Abstract: effectiveness] OR [Abstract: improve*] OR [Abstract: evaluat*] OR [Abstract: efficacy] OR [Abstract: accuracy] OR [Abstract: accurate]]	

[a]Searched on 13th December 2023

[b]ACM Digital Library does not use MeSH terms, so all MeSH terms were included as keywords
