# Supplementary material for: Evaluation of health recommender systems: a scoping review protocol
Source: BMJ Open. 2024 Oct 7;14(10):e083359. doi: 10.1136/bmjopen-2023-083359 (PMC11459321; doi:10.1136/bmjopen-2023-083359)
Supplement: Supplementary file 3 [file bmjopen-2023-083359.reviewer_comments.pdf]

PEER REVIEW HISTORY

BMJ Open publishes all reviews undertaken for accepted manuscripts. Reviewers are asked to complete a checklist review form and are provided with free text boxes to elaborate on their assessment. These free text comments are reproduced below.

ARTICLE DETAILS

**TITLE (PROVISIONAL)**	The Evaluation of Health Recommender Systems: A Scoping Review Protocol
**AUTHORS**	Ananthakrishnan, Ananya; Milne-Ives, Madison; Shankar, Rohit; Meinert, Edward

VERSION 1 - REVIEW

**REVIEWER NAME**	Ramis, Mary-Anne
**REVIEWER AFFILIATION**	Mater Health Services Brisbane, Mater Health and the Queensland Centre for Evidence Based Nursing and Midwifery: A JBI Centre of Excellence
**REVIEWER CONFLICT OF INTEREST**	I am an associate editor for JBI Evidence Synthesis journal and am on the editorial team for BMC Nursing.
**DATE REVIEW RETURNED**	03-Jan-2024

**GENERAL COMMENTS**	Thank you for the opportunity to review this manuscript which presents a protocol for a scoping review on health recommender systems.<br>The protocol is well presented and provides a framework for an interesting and useful review. The search strategy looks thorough, and I note the inclusion of the PRISMA-ScR checklist.<br>My only concern is ensuring the review does not extend beyond the limits of a scoping review. The research questions regarding effectiveness and ‘factors influencing effectiveness of the existing HRS” may lead toward different synthesis methods. A scoping review will allow you to map these features as they are reported on in the literature but is not an appropriate method for providing evidence on effectiveness or aetiology.<br>If the overarching goal of the review is to map and characterise features of the reported literature on HRS’s, then a scoping review is appropriate. However, I would suggest rephrasing the research questions slightly to align to the acronym PCC which is more common for scoping reviews – Population, Concept, Context – as recommended here: Peters, M., Godfrey, C., McInerney, P., Khalil, H., Larsen, P., Marnie, C., Pollock, D., Tricco, A., Munn, Z. Best practice guidance and reporting items for the development of scoping review protocols. JBI Evidence Synthesis 20(4):p 953-968, April 2022. | DOI: 10.11124/JBIES-21-00242<br><br>If, however, the main goal is to determine effectiveness of these systems then I suggest using systematic review methods which will allow you to determine effectiveness, or a review of aetiology and risk for identifying causal factors influencing outcomes.

	These articles may be of use: Munn, Z., Peters, M.D.J., Stern, C. et al. Systematic review or scoping review? Guidance for authors when choosing between a systematic or scoping review approach. BMC Med Res Methodol 18, 143 (2018). https://doi.org/10.1186/s12874-018-0611-x<br>Tricco, A.C., Lillie, E., Zarin, W. et al. A scoping review on the conduct and reporting of scoping reviews. BMC Med Res Methodol 16, 15 (2016). https://doi.org/10.1186/s12874-016-0116-4

**REVIEWER NAME**	Berahmand, Kamal
**REVIEWER AFFILIATION**	Queensland University of Technology
**REVIEWER CONFLICT OF INTEREST**	yes
**DATE REVIEW RETURNED**	25-Feb-2024

**GENERAL COMMENTS**	This paper presents an interesting proposal a new survey of recommender system in health Recommender Systems. However, there is room for improvement before the manuscript can be accepted for publication. I recommend that the authors carefully revise the manuscript based on the following feedback:<br>1- The author should rewrite the abstract because it does not adequately describe your work.<br>2.The introduction should be revised to clearly present the main ideas and motivations behind the proposed research. Please ensure that the research question and motivation of the proposed study are clearly stated. It is important to cover the research gap adequately.<br>3. Add a table of used symbols in the paper to improve readability.<br>4. The authors should Comments on the relevance of the results, comparison of the approaches, critical review of the relevant literature<br>5. the author should add Open problems in the separation section<br>6.The Literature citation is not adequate, and the related work to recommender should be discussed:<br>1. A novel healthy food recommendation to user groups based on a deep social community detection approach<br>2. An Effective Explainable Food Recommendation using Deep Image Clustering and Community detection

VERSION 1 – AUTHOR RESPONSE

Reviewer	Reviewer Feedback	Author response	Action taken	Text changes
Reviewer 1	Thank you for the opportunity to review this manuscript which presents a protocol for a scoping review on health recommender systems. The protocol is well presented and provides a framework for an interesting and useful review. The search strategy	We thank the reviewer for the positive feedback about the protocol.<br><br>We appreciate the concern about the scope of the paper. We have reviewed the wording in the protocol and	Aims and research question amended, and wording changed throughout the paper	“What is the evidence derived from evaluations of existing digital health recommender systems?(...)”<br><br>Table 1

	looks thorough, and I note the inclusion of the PRISMA-ScR checklist.<br><br>My only concern is ensuring the review does not extend beyond the limits of a scoping review. The research questions regarding effectiveness and 'factors influencing effectiveness of the existing HRS" may lead toward different synthesis methods. A scoping review will allow you to map these features as they are reported on in the literature but is not an appropriate method for providing evidence on effectiveness or aetiology.<br><br>If the overarching goal of the review is to map and characterise features of the reported literature on HRS's, then a scoping review is appropriate. However, I would suggest rephrasing the research questions slightly to align to the acronym PCC which is more common for scoping reviews – Population, Concept, Context – as recommended here: Peters, M., Godfrey, C., McInerney, P., Khalil, H., Larsen, P., Marnie, C., Pollock, D., Tricco, A., Munn, Z. Best practice guidance and reporting items for the development of scoping review protocols. JBI Evidence Synthesis 20(4):p 953-968, April 2022. | DOI: 10.11124/JBIES-21-00242	understand that the word 'effectiveness' might be misleading considering that the overarching goal is, indeed, to map and characterise features of the reported literature with a focus on evaluations of HRSs and their findings. We have amended the introduction and aims to reflect this and structured the research question based on the PCC framework.	Table 1 amended to PCC instead of PICOS	

	If, however, the main goal is to determine effectiveness of these systems then I suggest using systematic review methods which will allow you to determine effectiveness, or a review of aetiology and risk for identifying causal factors influencing outcomes.<br><br>These articles may be of use: Munn, Z., Peters, M.D.J., Stern, C. et al. Systematic review or scoping review? Guidance for authors when choosing between a systematic or scoping review approach. BMC Med Res Methodol 18, 143 (2018). https://doi.org/10.1186/s12874-018-0611-x<br><br>Tricco, A.C., Lillie, E., Zarin, W. et al. A scoping review on the conduct and reporting of scoping reviews. BMC Med Res Methodol 16, 15 (2016). https://doi.org/10.1186/s12874-016-0116-4			
Reviewer 2	This paper presents an interesting proposal a new survey of recommender system in health Recommender Systems. However, there is room for improvement before the manuscript can be accepted for publication. I recommend that the authors carefully revise the manuscript based on the following feedback:<br><br>The author should rewrite the abstract because it does not adequately describe your work.	We appreciate the reviewer's suggestion to revise the abstract so that it better describes our work. We have now amended the abstract based on your feedback by providing more detail about health recommender systems in the 'Background' section and better explaining the search strategy and data analysis in the 'Methods and analysis' section.	Abstract amended	Background: People increasingly rely on online health information for their health-related decision-making. Given the overwhelming amount of information available, the risk of misinformation is high. Health recommender systems, which recommend personalised health-related information or interventions using intelligent algorithms, have the potential to address this issue. Many such systems

				have been developed and evaluated individually, but there is a need to synthesise the evaluation findings to identify gaps and ensure that future recommender systems are designed to have a positive impact on health or target behaviours.<br><br>Objective: The purpose of this review is to provide an overview of the state of the literature evaluating health recommender systems and highlight lessons learned, methodological considerations, and gaps in current research.<br><br>Methods and analysis: The review will follow the Preferred Reporting Items for Systematic Reviews and Meta-Analyses extension for Scoping Reviews (PRISMA-ScR), and the Population, Intervention, Comparator, Outcome, and Study (PICOS) frameworks will be used to structure this review. Five databases (PubMED, ACM Digital Library Full-Text Collection, IEEE Xplore, Web of Science, and ScienceDirect) will be searched for studies published in English that evaluate at least one health recommender system using search terms following the

				themes reflecting digital health, recommendation systems, and evaluations of efficacy and impact. After using EndNote 21 for initial screening, two independent reviewers will screen the title and abstracts and full texts of the references, and then extract data from included studies related to the recommender system characteristics, evaluation design, and evaluation findings into a predetermined form. A descriptive analysis will be conducted to provide an overview of the literature; key themes and gaps in the literature will be discussed.<br><br>Ethics and dissemination: Ethical approval is not required as data will be obtained from already published sources. Findings from this study will be disseminated via publication in a peer-reviewed journal.
Reviewer 2	The introduction should be revised to clearly present the main ideas and motivations behind the proposed research. Please ensure that the research question and motivation of the proposed study are clearly stated. It is important to cover the research gap adequately.	We thank the reviewer for their feedback about the introduction. We have now amended the structure of this section to better reflect the main ideas, motivations, and research gap.	Introduction amended and restructured	*[Full introduction with tracked changes is included in revised manuscript]*

Reviewer 2	Add a table of used symbols in the paper to improve readability.	We appreciate the reviewer's suggestion to add a table for clarity about symbols. To address this feedback, we have added clarification in two sections:<br><br>1) We have added a table in the main body of the manuscript listing the abbreviations and their expanded forms.<br><br>2) We have also added a footnote after Table 2 (Search Strings) to clarify the meaning of the asterisk (*) symbol used within the example search string.	Footnote and table added.	1) "Table 4. Abbreviations used in the text" added<br><br>2) Table 2. (Footnote) – "Note: * refers to the symbol commonly used by databases to indicate truncation or 'wildcard' searching, which has been used to indicate a search of all variants of the mentioned terms"
Reviewer 2	The authors should Comments on the relevance of the results, comparison of the approaches, critical review of the relevant literature	We thank the reviewer for their feedback regarding the relevance of the results, the comparison of approaches, and the critical review of the literature. However, as this is a protocol paper, BMJ Open guidelines discourage the discussion of expected findings at this stage. We have presented a brief explanation of the current literature in the 'Introduction' section; a more detailed discussion of the results and approaches will be included in the subsequent paper which will detail the findings of the review.	N/A	N/A
Reviewer 2	the author should add Open problems in the separation section	We are unclear what specifically the reviewer is suggesting here, as there is no section titled 'separation'. Regarding open problems in the literature, this is	N/A	N/A

		something that will be discussed in the full review, in line with BMJ Open guidelines not to include discussion of expected findings in review protocols.		
Reviewer 2	The Literature citation is not adequate, and the related work to recommender should be discussed:<br><br>1. A novel healthy food recommendation to user groups based on a deep social community detection approach<br><br>2. An Effective Explainable Food Recommendation using Deep Image Clustering and Community detection	We thank the reviewer for the suggestion of specific relevant literature to include. We have included the two papers in the Introduction section.	Citations added	N/A
